# The Formation of Glycan-Specific Natural Antibodies Repertoire in GalT-KO Mice Is Determined by Gut Microbiota

**DOI:** 10.3389/fimmu.2019.00342

**Published:** 2019-03-05

**Authors:** Daniel Bello-Gil, Christophe Audebert, Sara Olivera-Ardid, Magdiel Pérez-Cruz, Gaël Even, Nailya Khasbiullina, Nausicaa Gantois, Nadezhda Shilova, Sophie Merlin, Cristina Costa, Nicolai Bovin, Rafael Mañez

**Affiliations:** ^1^Infectious Pathology and Transplantation Division, Institut d'Investigació Biomèdica de Bellvitge, Barcelona, Spain; ^2^Genes Diffusion, Douai, France; ^3^PEGASE-Biosciences, Institut Pasteur de Lille, Lille, France; ^4^CNRS, INSERM, CHU Lille, Institut Pasteur de Lille, U1019-UMR 8204-CIIL-Centre d'Infection et d'Immunité de Lille, Université de Lille, Lille, France; ^5^Zelinsky Institute of Organic Chemistry, Moscow, Russia; ^6^Lille University, CNRS, Inserm, Lille University Hospital, Pasteur Institute of Lille, U1019 -UMR 8204 -CIIL-Centre d'Infection et d'Immunité de Lille, Lille, France; ^7^Shemyakin-Ovchinnikov Institute of Bioorganic Chemistry, Russian Academy of Sciences, Moscow, Russia; ^8^Intensive Care Department, Bellvitge University Hospital, Barcelona, Spain

**Keywords:** GalT-KO mice, gut microbiota, metagenetic high-throughput sequencing, 16S rRNA gene, natural anti-glycan antibodies, printed glycan array, metagenome-wide association studies

## Abstract

Gut commensal bacteria are known to have a significant role in regulating the innate and adaptive immune homeostasis. Alterations in the intestinal microbial composition have been associated with several disease states, including autoimmune and inflammatory conditions. However, it is not entirely clear how commensal gut microbiota modulate and contribute to the systemic immunity, and whether circulating elements of the host immune system could regulate the microbiome. Thus, we have studied the diversity and abundance of specific taxons in the gut microbiota of inbred GalT-KO mice during 7 months of animal life by metagenetic high-throughput sequencing (16S rRNA gene, variable regions V3–V5). The repertoire of glycan-specific natural antibodies, obtained by printed glycan array technology, was then associated with the microbial diversity for each animal by metagenome-wide association studies (MWAS). Our data show that the orders *clostridiales* (most abundant), *bacteriodales, lactobacillales*, and *deferribacterales* may be associated with the development of the final repertoire of natural anti-glycan antibodies in GalT-KO mice. The main changes in microbiota diversity (month-2 and month-3) were related to important changes in levels and repertoire of natural anti-glycan antibodies in these mice. Additionally, significant positive and negative associations were found between the gut microbiota and the pattern of specific anti-glycan antibodies. Regarding individual features, the gut microbiota and the corresponding repertoire of natural anti-glycan antibodies showed differences among the examined animals. We also found redundancy in different taxa associated with the development of specific anti-glycan antibodies. Differences in microbial diversity did not, therefore, necessarily influence the overall functional output of the gut microbiome of GalT-KO mice. In summary, the repertoire of natural anti-carbohydrate antibodies may be partially determined by the continuous antigenic stimulation produced by the gut bacterial population of each GalT-KO mouse. Small differences in gut microbiota diversity could determine different repertoire and levels of natural anti-glycan antibodies and consequently might induce different immune responses to pathogens or other potential threats.

## Introduction

Humans are colonized by trillions of microbial cells (1), the majority of this microbial ecosystem residing in the gut. The gut microbiome or gut microbiota (GM) is a very complex organ (2), its composition is dynamic (3, 4). GM has a profound primary influence on human nutrition (digestion and absorption of nutrients), and metabolism, and seems to play a critical role in the development and function of the host immune system (5). The microbiome regulates the immune system at the mucosal level by producing active metabolites (1). The physiological interaction between the host immune system and the GM is important for preventing tissue-damaging inflammatory responses directed against commensals while avoiding infection by pathogens or the uncontrolled growth of indigenous pathobionts (3). Alterations in composition and function of human GM have been associated with several pathologies, including metabolic disorders such as type-2 diabetes (6), obesity (7); cardiovascular diseases (8); autoimmune diseases such as inflammatory bowel disease (9), type-1 diabetes (10); cancer (11), and diseases related to the central nervous system like Alzheimer's and Parkinson's diseases (12), and multiple sclerosis (13). The interplay between the immune system and GM is very complex, and the underlying molecular mechanisms of host-microorganism interactions remain largely unknown (14).

One of the circulating elements of the immune system that seems to be closely related to GM development are the natural antibodies (NAbs). Little is known about factors involved in the regulation of the repertoire of NAbs (15). They are spontaneously produced by B-1 cells from early-stage of life, without any previous external immunological stimulation (16, 17). Their levels and binding affinities remain almost constant during the lifetime (18). Most of these antibodies target carbohydrate structures and its origin, repertoire, and physiological role are still controversial (19). The most accepted origin hypothesis suggests that stimulation of B-1 lymphocytes is produced by exposition to antigenic determinants of the gut microbiota (20). The differences observed in the composition of circulating anti-glycan NAbs in BALB/c mice (15), also reflect the uncertainties about the physiological role and origin of these antibodies. Nevertheless, increasing evidences describe the functional involvement of anti-glycan antibodies in different immunological mechanisms both in health and disease (21–24).

In humans, NAbs include xenoantibodies that react to galactose α1-3 galactose (αGal) epitopes. Primates, including humans, apes, and Old World monkeys, produce these antibodies. They do not express the αGal epitopes due to the inactivation of the gene coding for the α1,3-galactosyltransferase enzyme (25, 26). Natural anti-αGal antibodies are mainly known for being responsible for the initial rejection of mammalian xenografts exposing this structure (27, 28). One of the animal models more often used to study these antibodies are mice in which the gene coding for the α1,3-galactosyltransferase enzyme has been knocked out (GalT-KO). GalT-KO mice naturally produce antibodies directed to galactose α1-3 galactose (αGal) epitopes (anti- αGal antibodies) (29), one of the most common circulating anti-glycan antibodies found in humans.

Previous studies have shown that the repertoire of anti-carbohydrate NAbs appears to be not the same in genetically identical BALB/c mice (15). Additionally, no circulating anti-glycan antibodies was found in Swiss Webster mice born and housed under sterile conditions (30). Although results from animal models are not always translatable to humans and conclusions should be made with caution (31), this work is aimed to study the origin of natural circulating anti-carbohydrate antibodies by GM stimulation in GalT-KO mice during the first 7 months of life. The repertoire of circulating anti-carbohydrate antibodies of GalT-KO mice will be studied by Printed Glycan Array (PGA), and gut microbiota analysis will be carried out on fresh animal feces by high throughput sequencing.

## Materials and Methods

### α1,3-galactosyltransferase Knocked Out Mice

This study was performed with mice (*n* = 11, 9 male and 2 female) in which the gene coding for the α1,3-galactosyltransferase enzyme had been knocked out, and were derived from a highly inbred colony with a hybrid genetic background (B6xCBAx129sv) (29). Mice were weaned at 3 weeks, and then maintained individually in separated cages during 7 months at the IDIBELL animal facility (specific pathogen-free, SPF) under controlled temperature (21 ± 1°C), humidity (55 ± 5%) and cycles of light/dark (12/12 h). Food and water were given *ad libitum*. Teklad global 14% protein (Envigo, Huntingdon, UK) was used as a standard rodent maintenance diet.

### Feces and Serum Collection and Processing

Mouse body weight was measured from month 1 to 7 of the animal life. Feces were monthly obtained under restrain by letting the mouse defecate directly into autoclaved 1.5 ml tubes to avoid cross-contamination. The tubes were immediately placed in dry ice and stored at −80°C for further analysis. Mouse blood collection was also performed at 3 weeks (after weaning) and then every month by submandibular bleeding without anesthesia (32). Serum was collected by mild centrifugation (10 min, 1,200 g at 4°C) and stored at −80°C until further analysis.

### DNA Extraction and Quantification

Total genomic DNA was extracted directly from 50 mg of mouse fecal samples using the FastDNA^®^ SPIN Kit for Soil (Fps) (MP Biomedicals, USA) (33), according to the manufacturer's recommended procedures. DNA was eluted in 100 μl of elution buffer and stored at −20°C. Total DNA concentration was measured using the Quant-iT PicoGreen dsDNA assay (Invitrogen, Carlsbad, CA, USA).

### Metagenetic High-Throughput Sequencing

The sequenced regions of the 16S rRNA gene spanning variable regions V3–V5 were amplified using the broad-range forward primer For16S_519, CAGCMGCCGCGGTAATAC and the reverse primer Rev16S_926, CCGTCAATTCMTTTGAGTTT, as we previously described (34).

### Quantification of Anti-αGal Antibodies by ELISA

Nunc MaxiSorpTM 96-well flat bottom plates (Thermo Fisher Scientific, Waltham, MA, USA) were coated overnight at 4°C with 2.5 μg/mL of Galα1-3Galβ1-4GlcNAc glycan conjugated to human serum albumin (HSA; Dextra Laboratories, Reading, Berkshire, UK) in coating buffer (0.05 M carbonate-bicarbonate, pH 9.6). Wells coated only with 2.5 μg/mL of HSA (Sigma-Aldrich, St. Louis, MO, USA) were considered the background of each serum sample assessed. After coating, the plates were washed three times with PBS with 0.5% (v/v) Tween-20 (Sigma-Aldrich, St. Louis, MO, USA), and then blocked for 1 h at 4°C with 0.05% (v/v) Tween-20 in PBS. Washing steps were repeated, and serum samples diluted in PBS (1:100 for IgM and IgG, and 1:25 for IgA) were added to the wells and incubated for 1 h at 25°C. After washing, the plates were incubated for 1 h at 25°C with horseradish peroxidase (HRP)-labeled secondary antibodies diluted in PBS; 1:4,000 for goat anti-mouse IgM, IgG, and IgA (Invitrogen, Carlsbad, CA, USA). After another round of washing, an HRP substrate (o-phenylenediamine tablet sets, Sigma-Aldrich, St. Louis, MO, USA) was added to the wells, and the plates were incubated for 10 min at 25°C. The reaction was stopped with 3N HCl, and the resulting absorbance was registered at 492 nm using a microplate reader (BioTek, Winooski, VT, USA).

### Glycan Array Analysis

Glycan arrays (Semiotik LLC, Moscow, Russia) contained a collection of 682 amine-functionalized glycans (50 μM) and bacterial polysaccharides (10 μg/ml) printed onto N-hydroxysuccinimide derivatized glass slides (slide H, Schott-Nexterion, Mainz, Germany) as described before (35) at 6 replicates each. Synthetic glycan structures (>95% purity) are structurally identical the same as natural ones. NMR data of polysaccharides and related references are available in http://csdb.glycoscience.ru/bacterial. A complete list of the printed ligands can be found in the supplementary material (Supplementary Table 1). The binding results for IgM+IgG+IgA were expressed in RFU as the median ± IQR (25–75th). The step-by-step protocol was deposited in Protocol Exchange (36).

### Metagenome-Wide Association Studies (MWAS)

The reads obtained by metagenetic high-throughput sequencing were grouped into clusters (based on their similarities) and assigned taxonomically thanks to the Greengenes database v13.8 (https://greengenes.lbl.gov). Thus, for each mouse, we got a list of taxa and associated abundance. The totality of the taxa and their relative abundance in each mouse allowed calculating an alpha-diversity score and the microbial diversity at the individual level. The anti-glycan antibody data were compared with microbial diversity (and abundance) for each individual, to set associations and identify anti-glycan antibodies that potentially correlate with a specific taxon.

### Statistics

In PGA studies all glycans were printed in 6 replicates, and the binding results were expressed in relative fluorescence units (RFU) as the median and interquartile range (IQR, 25–75th). In the case of the ELISA, samples were determined by triplicate and levels of anti-αGal antibodies were expressed in relative units of Optical Density (OD) at 492 nm, as Mean ± SD. GraphPad Prism statics software (GraphPad Software Inc., San Diego, CA, USA) was used for analysis and data graphing.

To study microbial diversity in stool samples, Chao1 Diversity estimator was used with ANOVA post-tests and expressed as median (IQR, 25–75th). Spearman rank test was used to study the anti-glycan antibodies vs. microbiota interaction. The prediction of functional profiles from metagenomic linked to natural anti-glycan antibodies for GalT-KO mice was made using the Tax4Fun R package.

## Results

### Repertoire of Circulating Anti-carbohydrate Antibodies in GalT-KO Mice

**Figure 1:**
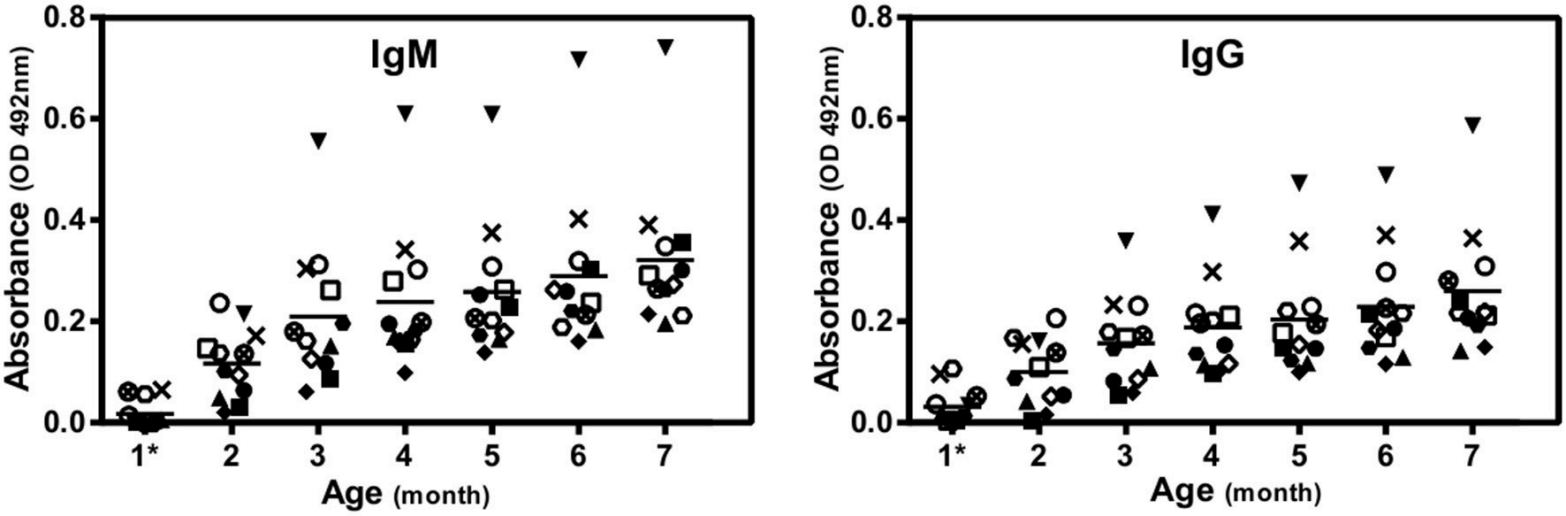
The levels of anti-αGal antibodies are variable between genetically identical GalT-KO mice. IgM and IgG were determined by ELISA and expressed in absorbance units (OD 492 nm). Each mark corresponds to a different mouse and represents the arithmetic mean of three replicates. The short horizontal line represents the arithmetic mean resulting from these determinations (*n* = 11). ^*^3 weeks of age.

The animal model used in this study spontaneously produced high blood levels of anti-αGal antibodies due to the inactivation of the enzyme α1,3-galactosyltransferase (Figure 1). Circulating levels of anti-αGal IgA antibodies were undetectable by ELISA.

After animal weaning, the baseline levels of natural anti-αGal antibodies were negligible. Later on (from month 2) mice started to produce anti-αGal antibodies. The highest increase in the anti-αGal concentration relative to recently weaned mice was attained at month 3 of life. Although there were slight increments in antibody concentration after this month, a plateau effect was observed, indicating that levels remain almost constant for the rest of the lifespan. Furthermore, these levels were variable between genetically identical mice, which were maintained from weaning in separate cages under identical housing conditions.

The repertoire of natural anti-carbohydrate antibodies in five male GalT-KO mice was also studied by printed glycan array (PGA) technology using a library of 682 different glycan structures (Supplementary Table 1). Most glycans were synthesized as –CH_2_CH_2_CH_2_NH_2_ spacer-armed O-glycosides, in several cases as –CH_2_CH_2_NH_2_ or –NHCOCH_2_NH_2_ glycosides. All glycan structures were characterized by high resolution (700 or 800 MHz) NMR spectroscopy, purified and tested by HPLC, indicating their >95% purity. The carbohydrates used in the PGA were structurally to the natural ones. In the PGA we considered values above 4,000 Relative Fluorescence Units (RFU) as a positive signal of antibody binding (this value is ~10% of the top glycans RFU), which were expressed as the median ± IQR. Due to constraints on mouse serum availability (~50 μl of serum per slide), the binding signal for a single glycan was the result of the contribution of IgM, IgG, and IgA together.

**Figure 2:**
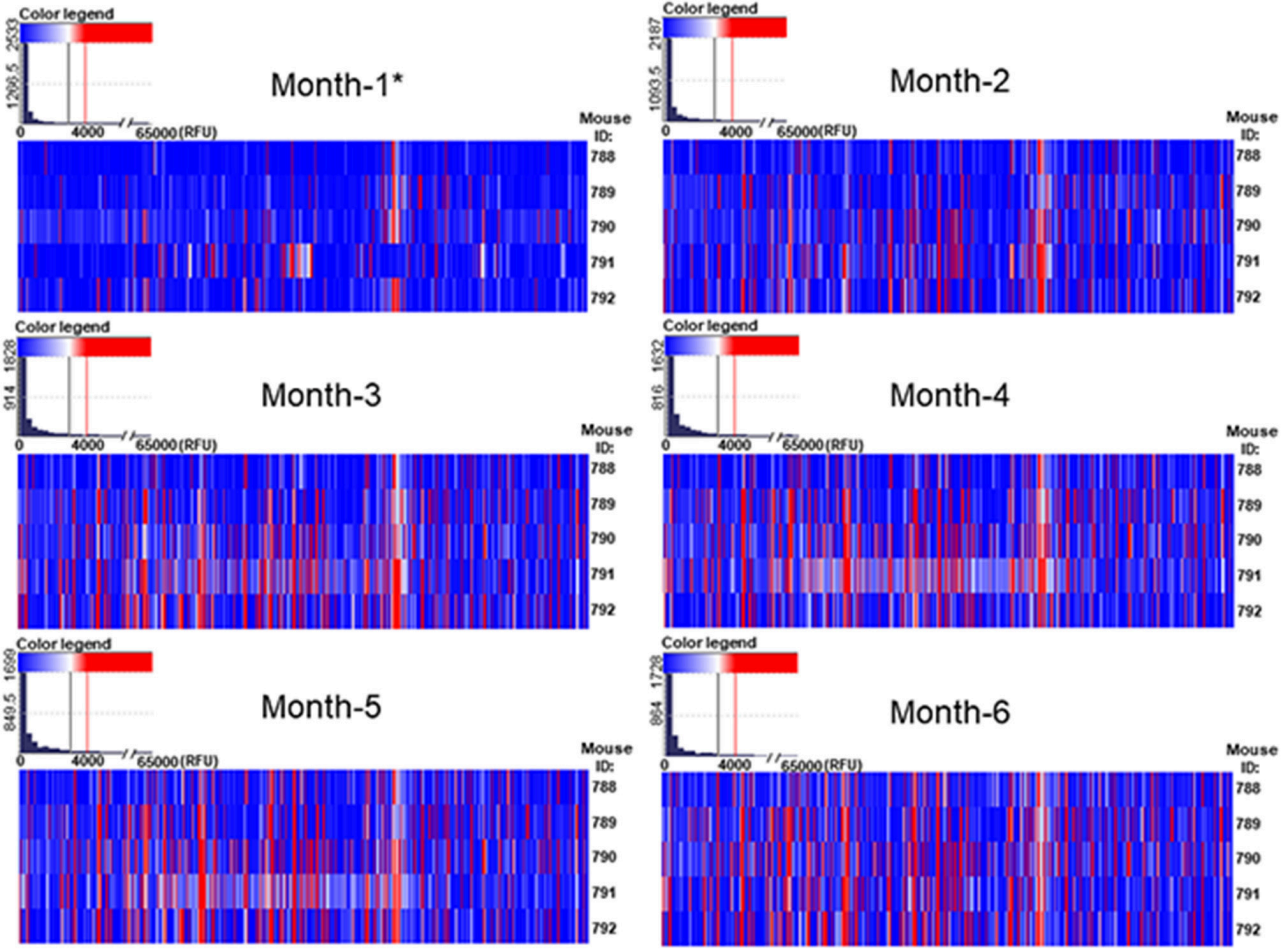
The repertoire of natural circulating anti-carbohydrate antibodies in GalT-KO is a function of age. GalT-KO mouse (*n* = 5) serum (1:15) was incubated with chips printed with 682 different glycans (6 replicates). Chips were scanned using a ScanArray GX Plus reader and data were analyzed with the ScanArray Express Microarray Analysis System (PerkinElmer). The binding results for IgM+IgG+IgA were expressed in RFU as the median ± IQR (25–75th). In the heat map, blue and white colors represent binding signals in RFU lower than 4,000 (background); red color signals ≥ 4,000 RFU (positive binding). ^*^3 weeks of age.

We have detected that the majority of printed glycans were not recognized by any natural antibody present in the serum of GalT-KO mice (Figure 2, in white and blue). The repertoire of natural anti-carbohydrate antibodies was quite limited at month 1 of life (Figure 2, in red). Only 13 out of 682 printed glycan structures (~2%) were recognized by NAbs present in the serum of at least 60% of the examined GalT-KO mice (Table 1).

**Table 1:** GalT-KO mice have a limited repertoire of NAbs after weaning.

**GID**	**Structure**	**Median (in RFU)**
60	6-O-Su-Galβ-sp	23,003
392	GalNAcα1-3(Fucα1-2)Galβ1-3GalNAcα-sp	22,042
1309	-[4Qui3NFoβ1-3Galα1-3GlcAβ1-3GalNAcβ1]_n_-	14,093
437	GalNAcα1-3(Fucα1-2)Galβ1-3GalNAcβ-sp	13,993
341	Neu5Acα2-3-(6-Su)Galβ1-4GlcNAcβ-sp	12,936
1220	-[2Fuc3(65%)Ac4(35%)Acα1-2Galβ1-3GalNAcα1-3GalNAcα1]_n_-	10,753
913	APPAT(Lac)YGPAPRTDPASTVGHAP-sp	9,672
914	APPATSGPAPRTDPASTVGHAP-sp	8,254
912	APPA(Tn)TSGPAPRTDPASTVGHAP-sp	6,252
320	4-O-Su-Neu5Acα2-3-(6-O-Su)Galβ1-4GlcNAcβ-sp	5,800
1208	-[4(R-Lac2-3Rha2Acα1-3)Manβ1-4Manα1-3GalNAcβ1]_n_-	5,064
5	GalNAcα-sp	4,782
177	3-O-Su-Galβ1-4(6-O-Su)GlcNAcβ-sp	4,639

*List of glycans with binding signals above 4,000 RFU at month 1 of life in at least 60% of examined mice (n = 5). sp means aminoethyl, aminopropyl, or glycyl spacer. Structures of polysaccharides are shown in “glycan form” instead of conventional “polysaccharide form” for the convenience of comparison with oligosaccharides; it means that we omitted designations of ring size (pyranose/furanose) and in most cases configuration (D/L). Accordingly, monosaccharides are pyranoses on default; fucose and rhamnose are L-sugars (on default), all others are D-sugars*.

From month 1 to month 2 the repertoire of natural anti-carbohydrate antibodies start gaining diversity since 41 glycans structures (~6%) were highly recognized by the sera of at least 60% of GalT-KO mice at month 2 of life (Figure 2, Table 2). Most of NAbs present in the examined animals at month 1 (77%) were also detected at month 2 but in a much higher concentration (Tables 1, 2). In fact, most of the glycans identified in month 1 appear as top rank glycans at month 2. On the contrary, only two NAbs were absent at month 2 that were detected at month 1: GID 5 and 913 (Tables 1, 2).

**Table 2:** GalT-KO mice gain diversity in the repertoire of natural anti-carbohydrate antibodies from month 2 of life.

**GID**	**Structure**	**Median (in RFU)**
392	GalNAcα1-3(Fucα1-2)Galβ1-3GalNAcα-sp	63,247
60	6-O-Su-Galβ-sp	39,239
2210	-[2Rha3(60%)Ac4(30%)Acα1-2Rhaα1-4GalAβ1-3GalNAcβ1]_n_-	37,928
437	GalNAcα1-3(Fucα1-2)Galβ1-3GalNAcβ-sp	37,618
2209	-[2Rha3(%)Ac4(%)Acα1-2Rhaα1-4GalAβ1-3GalNAcβ1]_n_-	35,047
1208	-[4(R-Lac2-3Rhap2Acα1-3)Manβ1-4Manα1-3GalNAcβ1]_n_-	28,422
271	Galβ1-6Galβ1-4Glcβ-sp	20,300
1220	-[2Fuc3(65%)Ac4(35%)Acα1-2Galβ1-3GalNAcα1-3GalNAcα1]_n_-	19,868
1309	-[4Qui3NFoβ1-3Galα1-3GlcAβ1-3GalNAcβ1]_n_-	19,539
320	4-O-Su-Neu5Acα2-3-(6-O-Su)Galβ1-4GlcNAcβ-sp	19,459
1610	-[3LQuiNAcα1-3GlcNAcα1-6(S-Lac-1-3)GlcNAcα1]_n_-	18,963
396	(GlcNAcβ1)_3_-3,4,6-GalNAcα-sp	16,860
256	GlcNAcβ1-6(GlcNAcβ1-4)GalNAcα-sp	15,831
404	GalNAcα1-3Galβ1-4(Fucα1-3)GlcNAcβ-sp	15,141
117	GlcNAcβ1-4GlcNAcβ-sp	14,679
375	Galα1-4GlcNAcβ1-3Galβ1-4GlcNAcβ-sp	12,664
2105	GalNAcA3Ac6NH2α1-4GalNAcAα1-3GlcNAc	12,081
362	Galα1-3(Fucα1-2)Galβ1-3GalNAcα-sp	11,541
913	APPAT(Lac)YGPAPRTDPASTVGHAP-sp	11,455
802	Galβ1-3GalNAc(fur)β-sp	11,344
1310	-[3GlcAβ1-4(Glcα1-3)Fucα1-4Fucα1-2Glcβ1-3GlcNAcα1]_n_-	10,825
154	3-O-Su-Galβ1-3GlcNAcβ-sp	10,322
914	APPATSGPAPRTDPASTVGHAP-sp	10,132
341	Neu5Acα2-3-(6-Su)Galβ1-4GlcNAcβ-sp	9,744
177	3-O-Su-Galβ1-4(6-O-Su)GlcNAcβ-sp	9,473
408	GlcNAcβ1-4(GlcNAcβ1-3)Galβ1-4GlcNAcβ-sp	9,132
115	GlcNAcβ1-4GlcNAcβ-Asn	8,735
13	GlcNAcβ-sp	8,599
1253	-[2Ribfβ1-4Galβ1-4GlcNAcα1-4Galβ1-3GlcNAcα1]_n_-	7,642
912	APPA(Tn)TSGPAPRTDPASTVGHAP-sp	6,184
918	APPAT(Tn)SGPAPR(Tn)TDPASTVGHAP-PEG3-K-PEG3-TALVVDDGVLNEENV-PEG3-NH-ethoxy-cyclobutene-1,2-dione	6,038
1242	-[4GlcAβ1-4(GlcNAcβ1-2)GlcAβ1-3GlcNAcα1]_n_-	6,034
202	6-O-Su-GalNAcβ1-4(6-O-Su)GlcNAcβ-sp	6,025
8009	-[4(R-Lac2-3Rhap2Acα1-3)Manβ1-4Manα1-3GalNAcβ1]_n_- LPS	5,992
150	3-O-Su-Galβ1-3GalNAcα-sp	5,811
2601	*Acetobacter methanolicus* LPS^*^	5,741
181	3,4-O-Su_2_-Galβ1-4GlcNAcβ-sp	5,737
915	APPAT(Tn)SGPAPRTDPASTVGHAPPATSG-sp	5,479
904	TPTPVNPSTAPAPAPTPTFAC-sp	5,451
919	APPAT(Tn)SGPAPR(Tn)TDPASTVGHAP-sp	5,140
359	Galα1-3(Fucα1-2)Galβ1-3GlcNAcβ-sp	4,825

List of glycans with binding signals above 4,000 RFU at month 2 of life in at least 60% of examined mice (n = 5).

* *- mannan, exact structure is not available*.

**Figure 3:**
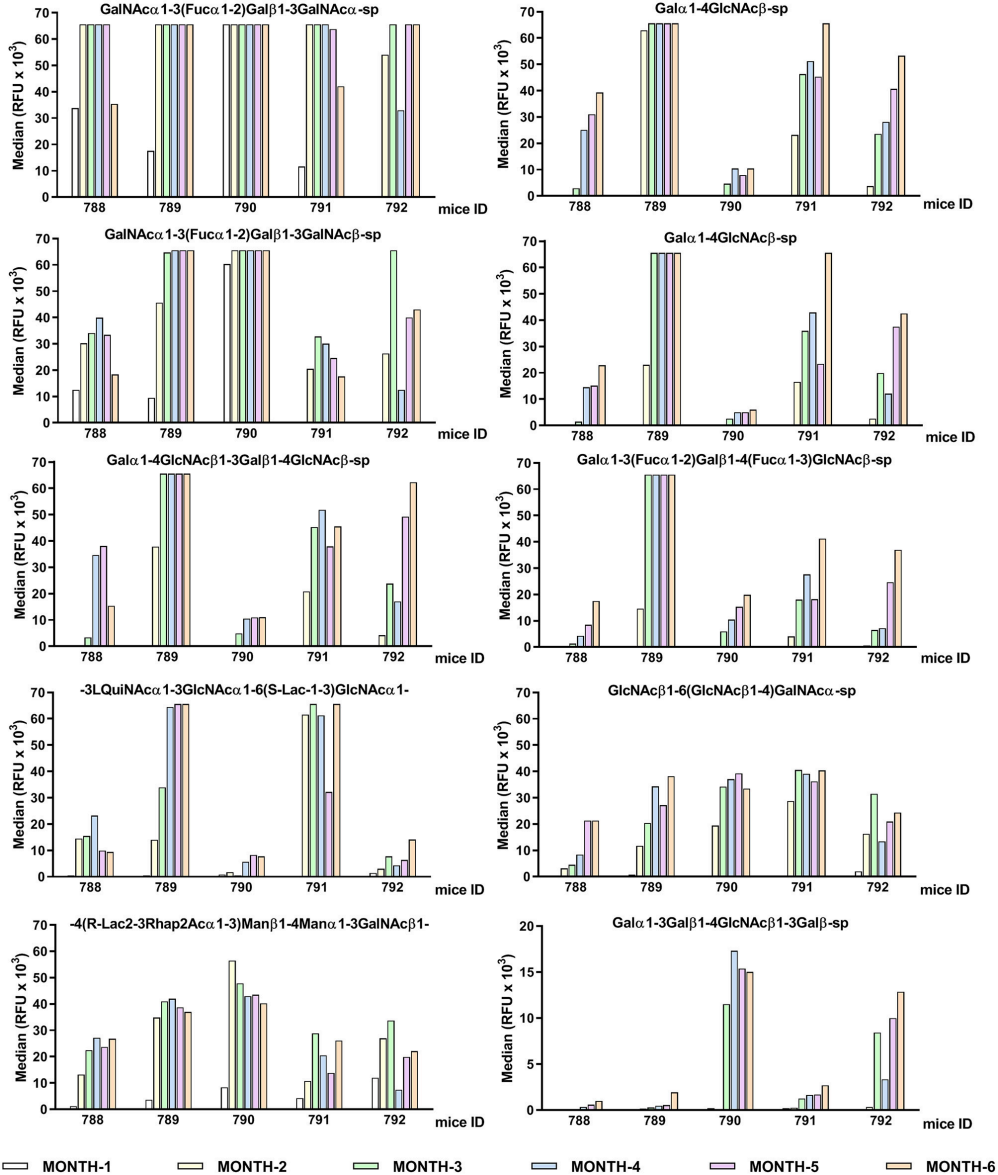
Individual differences in the conserved top-rank repertoire of circulating anti-glycan antibodies of GalT-KO mice during lifetime (*n* = 5). The first ten anti-glycan antibodies showing the strongest binding signals are represented. GalT-KO mice serum (1:15) was incubated with chips printed with 682 different glycans (6 replicates). Chips were scanned using a ScanArray GX Plus reader and data were analyzed with the ScanArray Express Microarray Analysis System (PerkinElmer). The binding results for IgM+IgG+IgA are expressed in RFU as the median.

As shown in Figure 2, the red color, signal of positive binding (≥4,000 RFU), gained more presence as the age of the animals increases, indicating a direct relation between mouse age and the diversity of the repertoire of natural anti-glycan antibodies. This behavior is evident when comparing month 1 to month 4 profiles. From month 4 up the repertoire shows minimal variation in diversity and quantity of most of the circulating anti-glycan antibodies (Figures 2, 3). At month 6 of age (adult mice), the pattern of natural anti-carbohydrate antibodies of GalT-KO mice comprised 78 different glycan-specificities.

The GalT-KO mice at month 6 of life shared 38% of the top-rank anti-glycan antibodies (≥10,000 RFU, Table 3). The rest of the top rank anti-glycan antibodies were produced by at least 60% of GalT-KO mice included in the study. Despite this high prevalence of the top-rank anti-glycan antibodies, each animal developed its specific background (Figure 3), showing differences in the individual repertoire and in the levels of natural anti-carbohydrate antibodies. NAbs targeting Galili-related structures (Table 3, in red) were within the top-rank circulating anti-glycan antibodies in GalT-KO mice. There was a significant spontaneous production of these antibodies, without any external antigenic stimulation, as a result of the inactivation of the gene coding for the α1,3-galactosyltransferase enzyme.

**Table 3:** The top-rank of conserved circulating anti-glycan antibodies among GalT-KO mice.

**GID**	**Structure**	**Median (in RFU)**
392	GalNAcα1-3(Fucα1-2)Galβ1-3GalNAcα-sp	54,803
82	Galα1-4GlcNAcβ-sp	46,800
437	GalNAcα1-3(Fucα1-2)Galβ1-3GalNAcβ-sp	42,008
81	Galα1-4GlcNAcβ-sp	40,463
375	Galα1-4GlcNAcβ1-3Galβ1-4GlcNAcβ-sp	39,947
483	Galα1-3(Fucα1-2)Galβ1-4(Fucα1-3)GlcNAcβ-sp	36,197
1610	-[3LQuiNAcα1-3GlcNAcα1-6(S-Lac-1-3)GlcNAcα1]_n_-	32,458
256	GlcNAcβ1-6(GlcNAcβ1-4)GalNAcα-sp	31,516
1208	-[4(R-Lac2-3Rhap2Acα1-3)Manβ1-4Manα1-3GalNAcβ1]_n_-	30,396
396	(GlcNAcβ1)_3_-3,4,6-GalNAcα-sp	28,387
408	GlcNAcβ1-4(GlcNAcβ1-3)Galβ1-4GlcNAcβ-sp	26,916
117	GlcNAcβ1-4GlcNAcβ-sp	26,220
364	Galα1-3Galβ1-4(Fucα1-3)GlcNAcβ-sp	26,162
1220	-[2Fuc3(65%)Ac4(35%)Acα1-2Galβ1-3GalNAcα1-3GalNAcα1]_n_-	21,307
362	Galα1-3(Fucα1-2)Galβ1-3GalNAcα-sp	18,088
13	GlcNAcβ-sp	17,559
181	3,4-O-Su_2_-Galβ1-4GlcNAcβ-sp	16,958
394	GlcNAcβ1-4(GlcNAcβ1-3)Galβ1-4GlcNAcβ-sp	10,652
373	Galα1-3Galβ1-4GlcNAcβ1-3Galβ-sp	6,681

*List of glycans with binding signals above 10,000 RFU at month 6 of life in all the examined mice (n = 5). GID 364, 372, and 483 contain the Galili epitope in their core structures (in red)*.

### Characterization of Gut Microbiota of GalT-KO Mice

The profile of gut microbial population was assessed by metagenetic high-throughput sequencing of GalT-KO mice fecal samples. Firstly, the weight of animals (*n* = 11) was followed for 7 months. The GalT-KO mice under examination showed a normal progression, achieving 80% of the total weight during month 2 and month 3 of life. At month 5, the majority of mice achieved their maximum weight, which remained almost constant until the end of the study (Supplementary Figure 1).

Bacterial DNA was extracted from fecal samples (feces), and Ion Torrent sequencing was performed by Gènes Diffusion (Institut Pasteur de Lille, France) to identify diversity in gut microbial population among the examined animals. High throughput sequencing data were deposited in the Sequence Read Archive (SRA) at NCBI (https://www.ncbi.nlm.nih.gov/sra/SRP132185). First, rarefaction curves (Supplementary Figure 2) were used to demonstrate that every fecal sample was rich enough in clustering high-throughput sequencing data to characterize the microbial community. This estimation was made with Chao1, which is a non-parametric richness estimator (37). The sample-based rarefaction curves (in colors) showed that GalT-KO feces present an adequate “species density” that is the number of taxa detected per sample.

**Figure 4:**
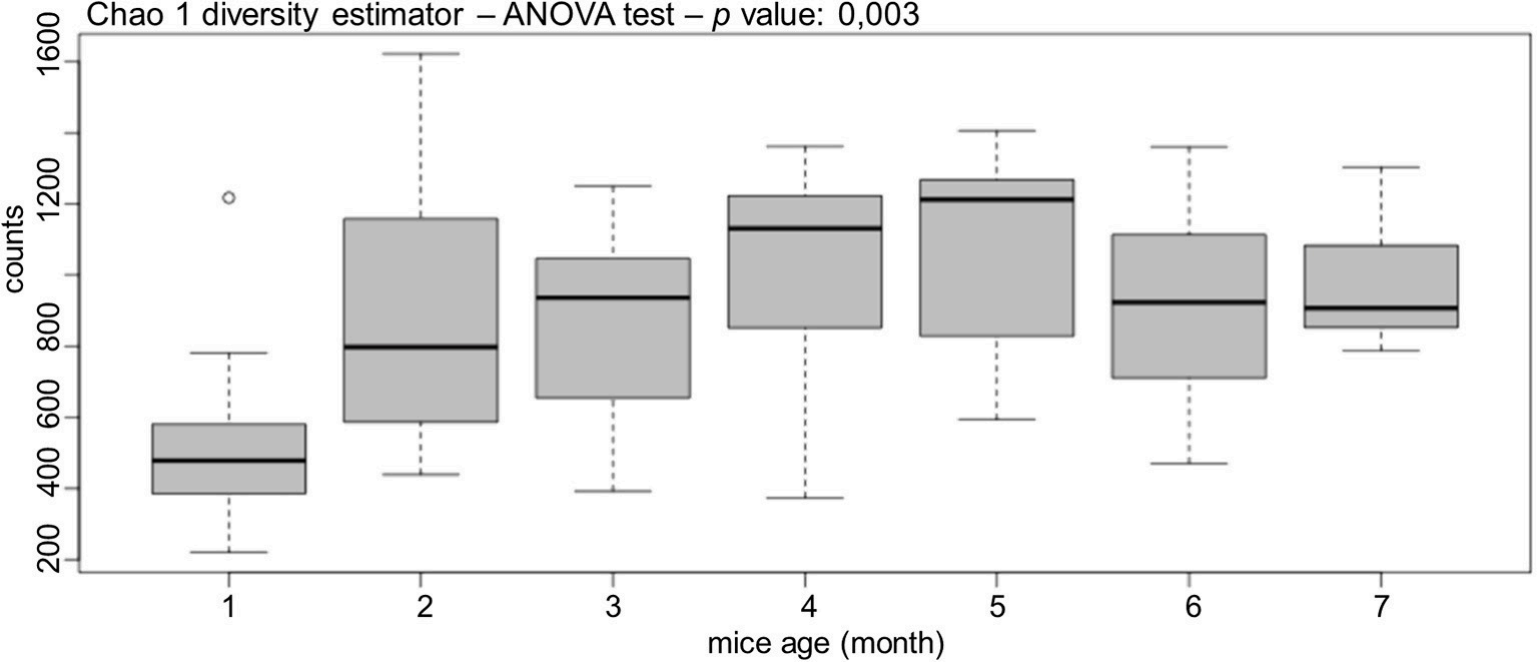
GalT-KO mice bacterial gut population is dynamic. The diversity of gut bacterial population was estimated by months with Chao1 diversity estimator (*n* = 77).

After sequencing, the diversity of gut bacterial population was estimated with Chao1 diversity estimator (Figure 4), which is a parametric estimation used to compare more than two populations in a completely randomized design. Bacterial diversity suffered a significant increase mostly after the second month of life, and then this diversity increased slowly until month 5. Finally, it remained almost stable from month 6 to 7 (Figure 4). The Chao1 estimator demonstrated that gut bacterial population is dynamic, showing significant differences in diversity during mouse life (*p* = 0.003).

**Figure 5:**
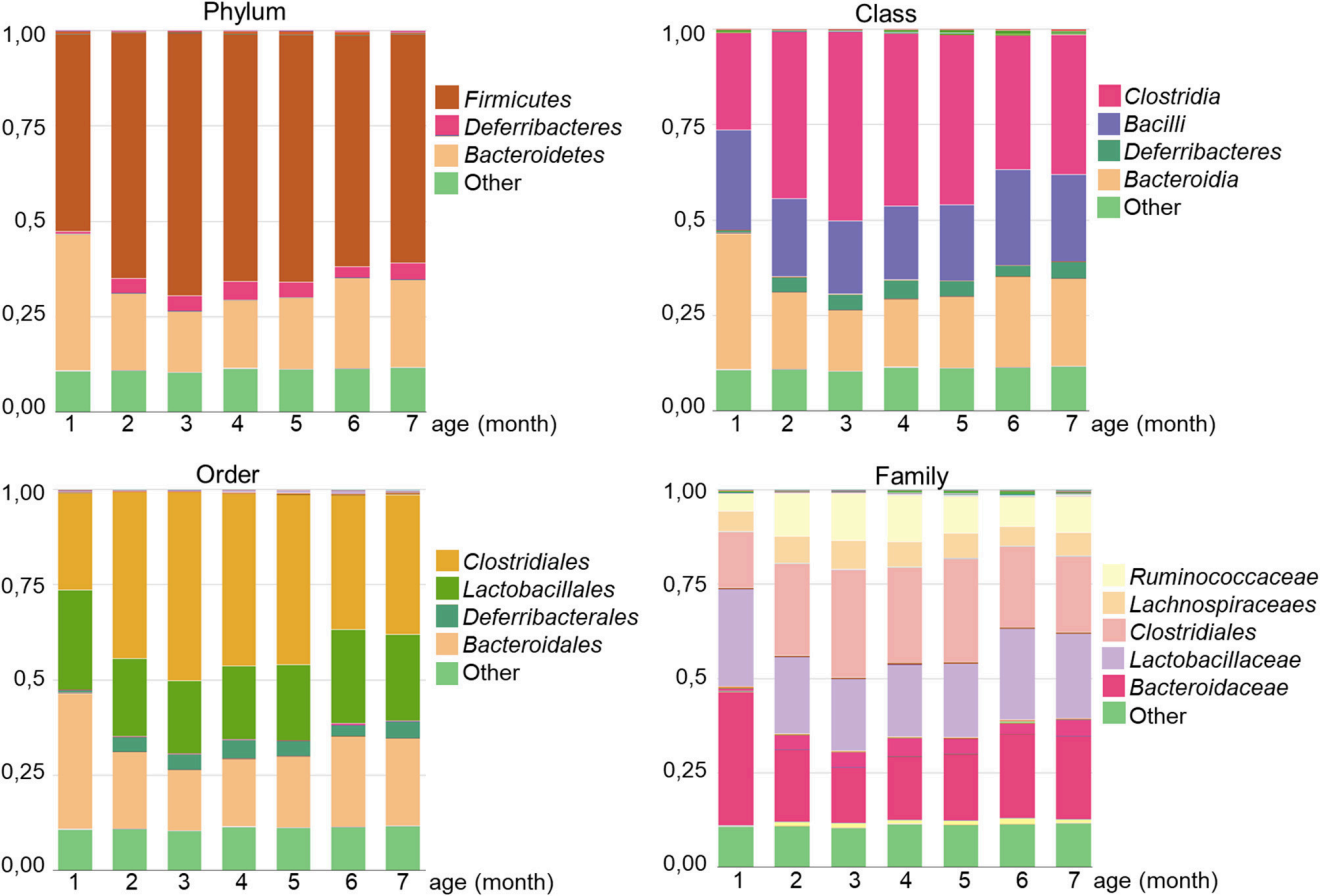
Analysis of gut microbiota diversity in GalT-KO mice during the first 7 months of life. The different microbial proportions corresponding to each phylum, class, order, and family are shown in colors (*n* = 77).

Regarding taxonomy, the regular gut microbiota of GalT-KO mice comprised two major phylum, namely *Firmicutes* and *Bacteroidetes* (Figure 5). At the class level, *Clostridia* was the main one, followed by *Bacilli* and *Bacteroidia* with similar proportions (Figure 5). *Clostridiales* was the predominant order, with *Lactobacilalles* and *Bacteroidales* showing very similar proportions. Finally, regarding family, *Bacteroidaceae* is the first family who colonized GalT-KO mouse gut. From month 2 to 7, the proportion of *Bacteroidaceae, Clostridiaceae*, and *Lactobacillaceae* (Figure 5) turned out to be very similar.

The differential expression analysis rendered significant differences in bacterial diversity and species richness between animals in the analyzed months. In the case, for example, of *Ruminococcus* (class *Clostridia*), a variation in relative abundance of this taxon is observed at month 1 among the examined mice (Supplementary Figure 3A). However, after month 3 of life, these differences were significantly reduced, showing a similar abundance between GalT-KO mice in this particular bacterial genus. The global analysis showed a dramatic increase in the counts of this taxon after 2 months of life, showing certain stability regarding taxon richness until the end of the study.

Similarly, within taxon *Mogibacteriaceae* (class *Clostridia*), a definite difference in relative abundance is observed at month 1 among the examined mice (Supplementary Figure 3B). These differences were also observed, contrary to *Ruminococcus*, during the whole study, where the relative abundance of this taxon was quite marked among animals. In general, it seems that although there are common microbiota populations among the examined animals, there also are differences in microbiota diversity and richness (abundance) in inbred animals maintained in different cages under identical housing conditions.

### Gut Microbiota Diversity and Glycan-Specific Natural Antibodies Repertoire Development

The ultimate goal of the present study is focused on setting associations between relative diversity and abundance of specific taxons with the development of specific patterns of natural anti-carbohydrate antibodies. This metagenome-wide association studies (MWAS), enables the high-resolution investigation of associations between immunological parameters like natural antibodies and gut microbiota. Taking this approach into account, we identified the production of a group of circulating anti-glycan antibodies significantly (*p* < 0.005) associated with the development of specific bacterial groups in the gut microbiota of individual GalT-KO mouse (summarized in Supplementary Table 2).

**Figure 6:**
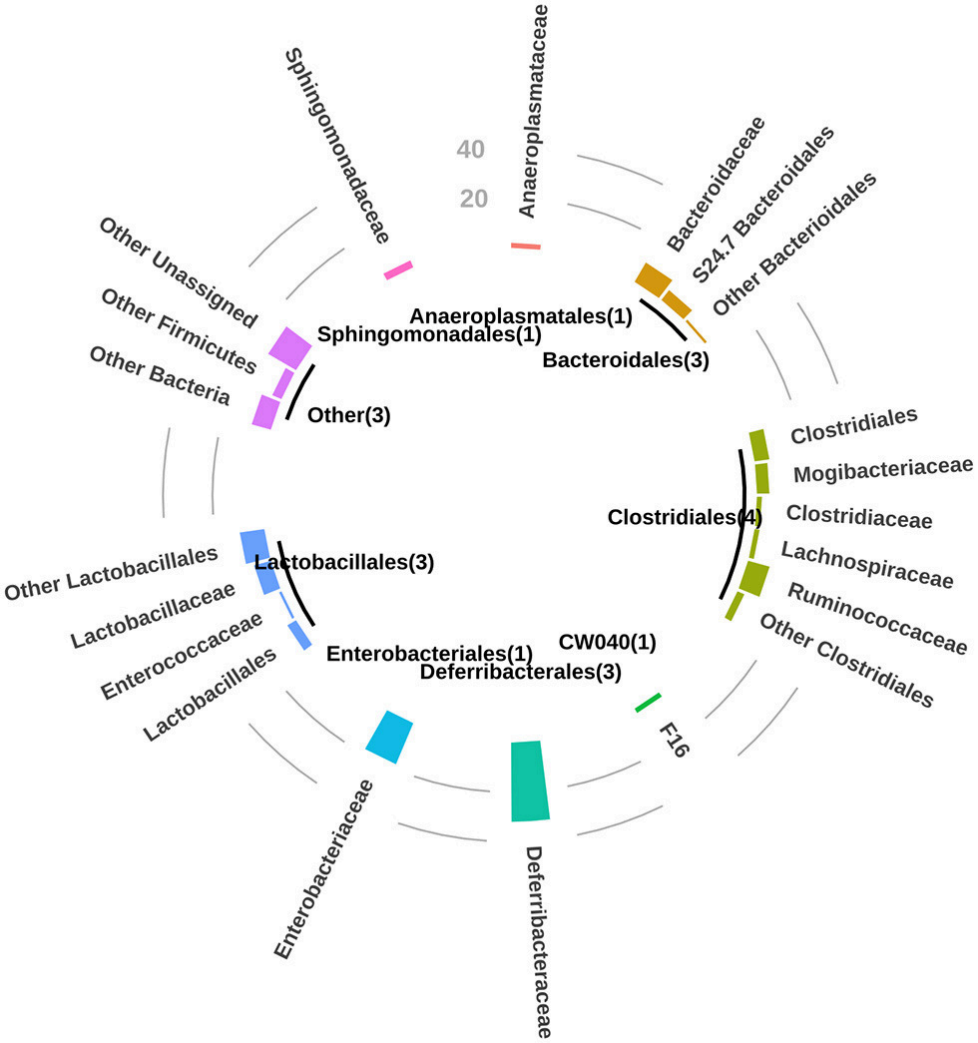
For *P*-value < 0.01, bar circle graph showing the number of natural anti-glycan antibodies structures associated with the bacterial taxa at a taxonomic level of order and family. Between brackets, in the central circle are the numbers of mice related to these observations.

A value of correlation of 1.0 means there is a perfect positive relationship between the two variables (taxon and anti-glycan antibody). For a positive increase in one variable, there is also a positive increase in the second one. A value of exactly −1.0 means there is a perfect negative relationship between the two variables, displaying opposite directions. In general, the orders *clostridiales* (most abundant), *bacteriodales, lactobacillales*, and *deferribacterales* were associated with the development of the repertoire of anti-glycan antibodies in GalT-KO mice (Figure 6).

The mentioned orders may thus regulate the production of natural anti-carbohydrate antibodies during the mouse lifetime. Additionally, and to a lesser extent among the examined animals, the orders *anaeroplasmatales* and *sphingomonadales* may be associated with the development of specific anti-glycan antibodies (Figure 6). The individual analysis of GalT-KO mice allowed us to find that not only one taxon was associated with one particular anti-glycan antibody since different taxa can trigger or regulate the production of the same anti-glycan antibody (Supplementary Table 2).

Additionally, the specific gut microbial population that induce or regulate the production of the repertoire of anti-glycan antibodies during the 7 months of GalT-KO mice, differed from one animal to another. Representative results of the association (positive or negative correlation) of taxon pattern with the regulation of the production of natural-specific anti-glycan antibodies are shown in Supplementary Figure 4. For example, the natural anti-glycan antibody directed toward GID #1805 (-4GalNAcβ1-3GlcNAcβ1-2(R-Lac1-3Glcα1-3)Rhaα1-2Ribfβ1-polysaccharide) negatively correlated with S24-7, a family of bacteria within the order *Bacteroidales*. Interestingly, as this family of bacteria disappeared from the microbiota, the level of these antibodies increased in the first 3 months of life. This profile changed from month 3 to month 6, presenting in fact the opposite effect. This alternancy (also observed for GID #316, #1259) might reflect a potential balance between the dominance of bacteria and natural antibodies. By contrast, natural anti-glycan antibodies targeting GID #202 and #1241, positively correlated with *clostridiales* and the family of *mogibacteriacea*, respectively. A positive increase in one of the mentioned variable was associated with a positive increase in the second one (Supplementary Figure 4 and Supplementary Table 2).

## Discussion

The present work studied the association between the diversity and colonization pattern of the gut microbiota and the development of the repertoire of natural anti-glycan antibodies in inbred GalT-KO mice. Our observations indicate that the influence of the gut microbiota on the quantity and repertoire of natural anti-glycan antibodies will probably be an additional mechanism by which the microbiota affect health and disease.

The microbiome constitutes the last human organ under active research. Like other organs, the individual health might be damaged when its collective population structure is altered (2, 38). Every day new direct and indirect physiological functions are attributed to microbiome, and more specifically to the gut microbiota, where microbial cells of thousands of taxonomic units are condensed (39). Since the intestinal tract is the main point of contact for the host immune system and commensal bacteria, microbiota plays a remarkable role in both local and systemic immune functions (40). Microbiota diversity and development, at the same time, is modulated by gut microenvironment. As gut is an anoxic area, the control of its oxidative stress and redox status could has a dramatic impact on gut microbial content, recently summarized elsewhere (41). More important, the haematopoietic and non-haematopoietic cells are located strategically at the host-microbiome interface (42). These cells act as anchors between the microbiota (and its metabolites) and the host immune-system, translating the signals into host physiological responses [reviewed elsewhere (42–44)]. Indeed, the metabolites derived from gut microbiota such as bacteriocins (45), short-chain fatty acids (43–45), quorum-sensing autoinducers (45), tryptophan, and retinoic acid metabolites (44) seem to be essential for intestinal homeostasis and maturation of immune system.

Microbial composition of the gastrointestinal (GI) tract is typically measured/isolated from fresh feces samples, which certainly does not truly reflect the full diversity of the GI tract (46). However, the methodology and results compiled here could be a valid approach to get relevant information about the composition of the whole GI tract. Additionally, this methodology allows the following of animals for a more extended period as feces collection is not an invasive and painful practice. Regarding the natural anti-carbohydrate repertoire, our results show that in early weaned mice (3 weeks) the repertoire of natural anti-glycan antibodies is quite limited, meaning that the immune system of young animals is at a very early stage of development. These antibodies detected at month-1 could be the result of passive immunization (47) through the placenta during the pregnancy (IgG) or via mother's milk (most of them are IgA), to protect the offspring from potential threats like bacterial infections during the first stage of life. The same general analysis applies to microbiota diversity; lactating mice don't need complex and diverse microbiota when milk is the primary dietary intake during the first month of life. After weaning, mice start incorporating to diet different new elements and, at the same time, gut microbiota is gaining in diversity and complexity. At this initial stage microbiota is formed by bacteria that pass to the animals during delivery, and direct colonization by bacteria present in the natural environment of animals. We've previously reported that there was no production of antibodies against oligosaccharides and bacterial polysaccharides in mice housed in condition excluding living bacteria. Sterile mice acquired an almost complete repertoire of anti-glycan antibodies once they were gavaged with a library of antigens from mice microbiota (30).

At month 2 of life mice presented the greatest differences (comparing consecutive months) regarding the diversity of gut bacterial population compare to weaned animals (month 1). After this period, both the repertoire of natural anti-carbohydrate antibodies and gut microbiota diversity increase slightly from month 3 to month 5 and then remain almost constant until month 7.

We have detected differences in the circulating levels of the conserved pattern of the natural anti-glycan antibodies among the examined animals. The rest of the circulating anti-glycan antibodies found in GalT-KO mice were randomly present in some animals and completely absent in others. We have shown similar results in BALB/c mice in a previous work, where we demonstrated the lack of identical repertoires of natural anti-carbohydrate antibodies between individual inbred mice (15). At month 6 of age, the pattern of natural anti-carbohydrate antibodies of single-housed GalT-KO mice comprised 78 different glycan-specificities. We described a similar diversity in the repertoire of anti-glycan antibodies in adult co-housed Balb/C (71 glyco-ligands) (15) and CD-1 mice (93 glyco-ligands) fed with standard granulated food (30). Thus, housing animals together in the same cage is not a critical factor to generate diversity in the repertoire of anti-glycan antibodies. Regarding feeding condition, nutrients have little influence on formation of natural anti-glycan antibodies repertoire as antigenic stimuli in nutrients is insufficient to prime natural antibodies (30). All these results together support the importance of early contact of the naive immune system with microorganisms of the animal environment to form the final repertoire of natural anti-glycan antibodies.

Regarding microbiota analysis, there are differences among the examined animals not only in the qualitative representation of taxa but notably in their quantitative contribution. Previous studies have shown that there are considerable differences in microbial composition between mouse strains mainly determined by animal provider and housing conditions (48–52). A significant divergence of the intestinal microbiota (microbiome variance) between founder and second generation of C57BL/6J mice strain, as well as continuing inter-generational variance, was also recently reported (53). Here we demonstrated that even inbred mice (same generation), maintained in individual cages under identical housing conditions, have differences in their gut microbiota.

Both global changes in the diversity of natural anti-glycan antibodies and gut microbiota, seem to be closely connected. The main changes in microbiota diversity (month 2 and month 3) were associated with important changes in levels and repertoire of natural anti-glycan antibodies. Regarding individual differences, both the gut microbiota and the repertoire of natural anti-glycan antibodies were not identical among the examined GalT-KO mice. We have previously demonstrated that genetically identical BALB/c mice should not be considered as “completely equivalent” from the immunological perspective as they present, despite some shared specificities, different profiles of circulating anti-glycan NAbs (15). Now we have demonstrated in GalT-KO mice that these differences may be determined by differences in the gut microbiota of individual animals. It is known that up to 90% of the immunoglobulin-secreting cells of the normal mouse gut produce NAbs that are completely absent in germ-free mice (54, 55). Non-pathogenic (commensal) bacteria possess millions of antigens, and they are capable to prime those B-1 lymphocytes genetically selected for the synthesis of natural antibodies (19, 56). Thus, “the appearance of a particular natural anti-carbohydrate antibody requires two keys - the existence of a B-1 cell gene and the priming with bacterial antigen (a mimotope of the cognate antigen). Bacteria are the best source for anti-carbohydrate antibody priming for two additional reasons: (1) appearance only after birth, (2) the need of toll-like receptors for recognition by B-1 cells; this mechanism excludes priming of B-1 cells with auto-antigens at the embryonic stage” (19).

We also found many significant positive and negative associations between the profiles followed by specific anti-glycan antibodies and gut microbiota. Remarkably, negative correlations may be indicative of a regulatory mechanism, a balancing between certain taxon abundance and the priming of a specific population of B-cells producing anti-glycan antibodies. In the case of positive correlations, it may reflect a causative effect with the appearance of one element triggering the development of the other one. Additionally, the specific gut microbial population that induce or regulate the production of the repertoire of anti-glycan antibodies during the 7 months of GalT-KO mice differed from one animal to another. This could be partially explained by the phenomenon of redundancy. We found that different taxa were associated with the development of one specific anti-glycan antibody. The gut microbiome is believed to contain substantial functional redundancy, with multiple bacterial taxa capable of contributing to similar metabolic outcomes (57). Therefore, differences in microbial diversity do not necessarily result in alterations in the overall functional output of the gut microbiome (53).

A recent study of co-authors of this paper partially support this finding. They demonstrated that specificity of the natural anti-glycan antibodies does not correspond to the chemical structure of carbohydrate antigens of specific bacterium orally inoculated to different groups of male Swiss Webster mice (30). Additionally, the priming with bacterial polysaccharide antigens led to the production of natural antibodies directed to completely structurally-different glycans of glycoproteins/glycolipids. This phenomenon of mimicry is expected for B1 cells, which generally produce polyreactive low affinity antibodies (30).

The gut microbiota development and diversity seem to be closely related to the early development of the innate immune system. The repertoire of anti-carbohydrate antibodies may be determined, at least in part, by continuos antigenic stimulation produced by the normal microbiota of each animal. This means that small differences in microbiota diversity could determine different repertoire of natural anti-glycan antibodies (30, 58). This might consequently produce different innate immune responses to pathogens or other potential threats. The microbiome development is probably essential for the animals immune system maturation.

The bilateral interaction between the host and its microbiota is very complex (30, 41–45). Although the presence or absence of a single bacterial species is not enough for understanding the detailed interaction between the microbiota and the host, here we provide evidences that support the important role of gut microbiota in the initial formation of the repertoire of natural anti-glycan antibodies. There is a significant association between these two elements, being the exposure to new antigens coming from commensal bacteria of gut microbiota the stimulation signal that triggers the production and regulation of part of the repertoire of natural antibodies. These results highlight the importance of the diversity and colonization pattern of the gut microbiota in the development of the individual repertoire of natural anti-glycan antibodies.

## Conclusions

Microbiota formation and diversity may partially orchestrate the production and final repertoire diversity of natural anti-carbohydrate antibodies in GalT-KO mice. Inbred animals maintained under identical housing conditions and separated in individual cages, despite some shared patterns, present differences in microbiota composition, and abundance. Consequently, the animals displayed a specific repertoire of natural anti-carbohydrate antibodies. We hypothetized the influence of the gut microbiota on the quantity and repertoire of natural anti-glycan antibodies is probably an additional mechanism by which the microbiota affect health and disease.

## Data Availability

High throughput sequencing data were deposited in the Sequence Read Archive (SRA) at NCBI (https://www.ncbi.nlm.nih.gov/sra/SRP132185).

## Ethics Statement

All animal procedures were supervised and approved by Bellvitge Biomedical Research Institute (IDIBELL) ethics committee for animal experimentation and the Catalonia Government. The care and handling of the animals conformed to the Guide for the Care and Use of Laboratory Animals published by the US National Institutes of Health (NIH Publication 85-23, revised 1996) and the European Agreement of Vertebrate Animal Protection for Experimental Use (86/609). The euthanasia procedure was established following the European Directive on the protection of animals used for scientific purposes (2010/63/EU). Briefly, the animals were placed in a transparent euthanasia chamber in which they were easily visible. The animals were euthanized by inhalation in an atmosphere of 100% carbon dioxide (CO_2_; flow rate of 50 L/min over 10 min) with early loss of consciousness and minimal pain, suffering, and distress. A maximum of five animals were introduced to the chamber at one time, allowing all animals adequate room to move.

## Author Contributions

DB-G designed all the experimental work, coordinated the study, made a substantial contribution to data management and analysis, and wrote the body of the manuscript. CA was in charge of metagenetic high-throughput sequencing and metagenome-wide association studies and made a substantial contribution to data analysis, and manuscript drafting. SO-A contributed with the experimental design, performed the experimental work related to animal samples collection, ELISA, and glycan array analysis, and participated in the manuscript drafting. MP-C made a substantial contribution to the design of the experiments and manuscript preparation. GE performed data analysis referred to high-throughput sequencing and metagenome-wide association studies and participated in the manuscript revision. NK participated in the glycan array analysis, data management, and presentation, and was involved in manuscript revision. NG performed DNA extraction. NS participated in the glycan array analysis, data presentation, and was involved in revising the manuscript critically. SM prepared the library and performed Ion Torrent Sequencing. CC maintained the colony of GalT-KO mice and made a substantial contribution to the final manuscript. NB supported part of this research, coordinated the glycan array analysis, and was involved in drafting the manuscript and revising it critically. RM supported and organized the whole study, made substantial contributions to conception and design, and was involved in writing the manuscript and editing it critically.

### Conflict of Interest Statement

The authors declare that the research was conducted in the absence of any commercial or financial relationships that could be construed as a potential conflict of interest.
