# Supplementary material for: The Formation of Glycan-Specific Natural Antibodies Repertoire in GalT-KO Mice Is Determined by Gut Microbiota
Source: Front Immunol. 2019 Mar 5;10:342. doi: 10.3389/fimmu.2019.00342 (PMC6411795; doi:10.3389/fimmu.2019.00342)
Supplement: Supplementary file 3 [file Data_Sheet_1.docx]

**Supplementary material**

**Supplementary Figure 1. GalT-KO mice showed a normal growth progression.** Animals were weighted monthly from weaning to month-7 of life. Results are expressed as arithmetic mean ± SD (n = 11).

**Supplementary Figure 2. Estimation of bacterial species density in samples of GalT-KO feces.** The sample-based rarefaction curves (in colors) represent the different months of feces collection (n = 77). The estimation was performed with Chao1, a nonparametric richness estimator. Mean number of reads / index 59176,73; sd number reads 19414,95; mean read length (bp) 266,26; sd read length (bp) 25,17; outlier (< 10000 reads) 0,00.

**Supplementary Figure 3. Individual and global analysis of the relative abundance (counts) of the taxon *Ruminococcus* (A) and *Mogibacteriaceae* (B) in gut microbiota of GalT-KO mice during 7 months of life (n = 11).**


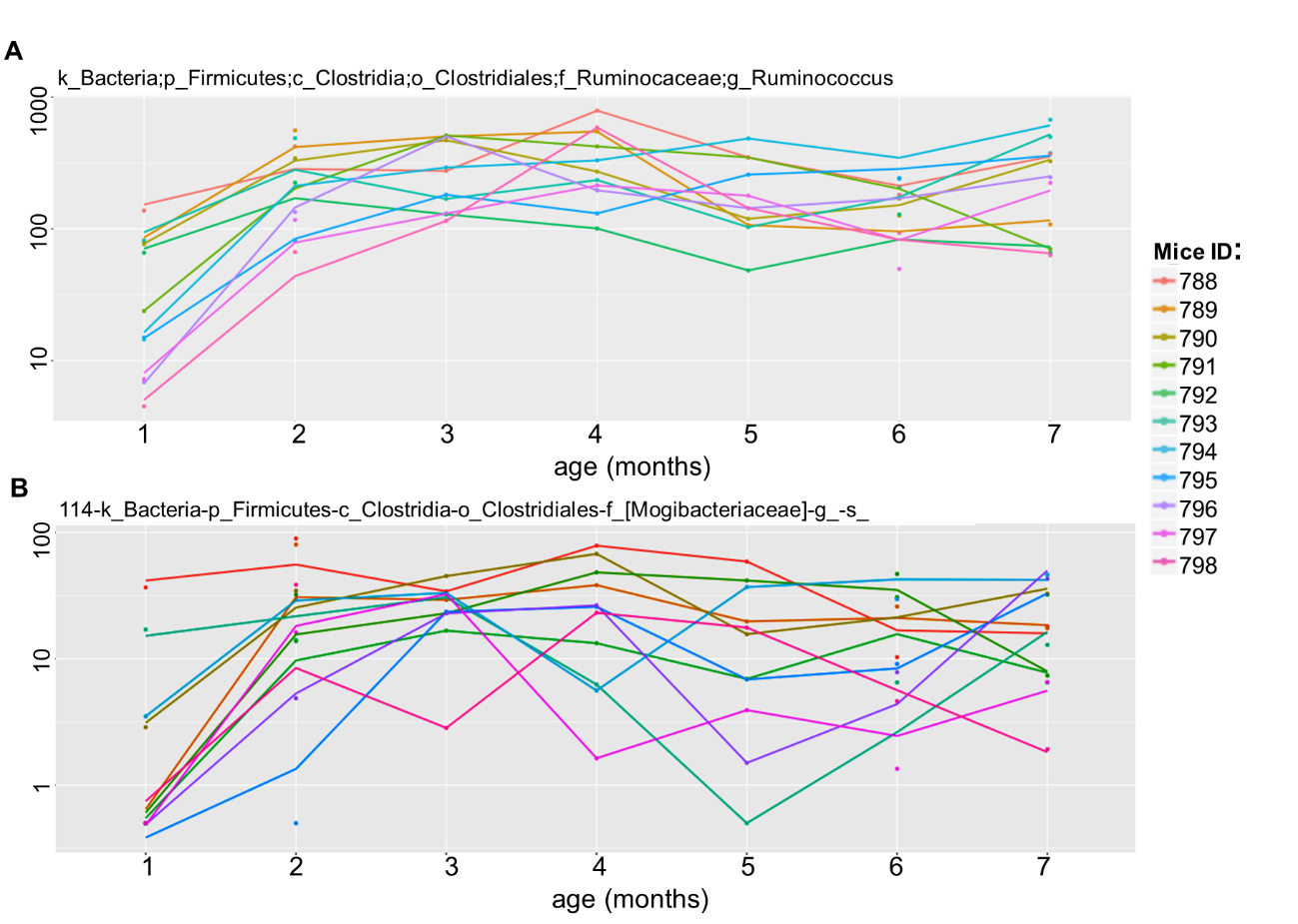


**Supplementary Figure 4. Representative results of the association (negative and positive correlation) of taxon trend (in yellow) with the the production of natural-specific anti-glycan antibodies (in dark orange) in GalT-KO mice.**


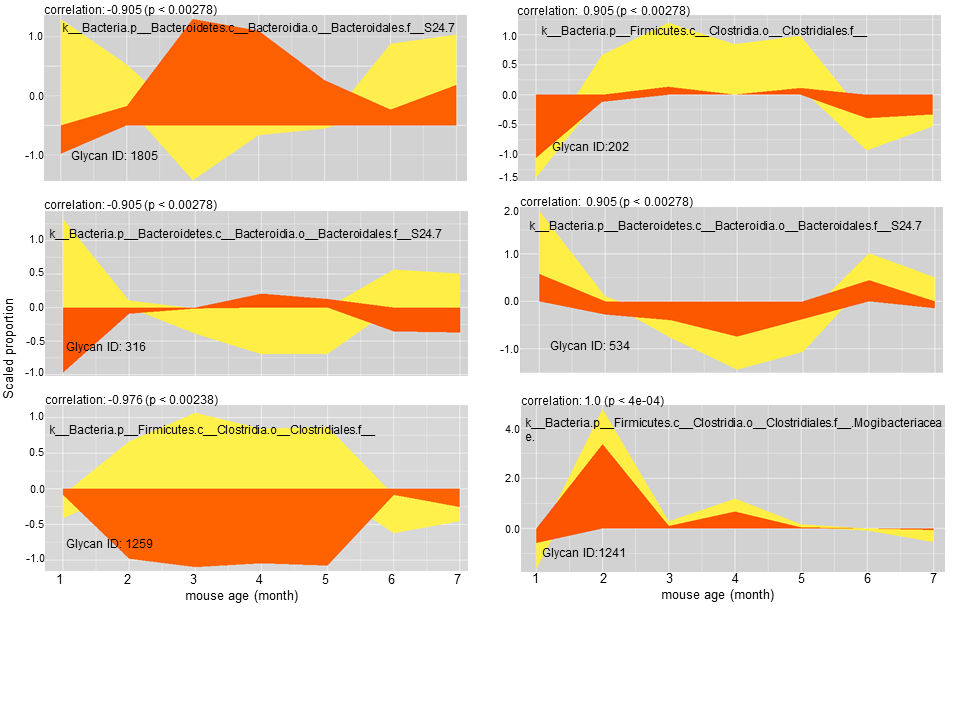


| Glycan ID | Structure |
| --- | --- |
| 1805 | 4GalNAcβ1-3GlcNAcβ1-2(R-Lac1-3Glcα1-3)Rhaα1-2Ribfβ1- (*Proteus vulgaris* O25) |
| 202 | 6-O-Su-GalNAcβ1-4(6-O-Su)GlcNAcβ-sp |
| 316 | Fucα1-2(6-Su)Galβ1-4GlcNAcβ-sp |
| 534 | Neu5Acα2-6Galβ1-4GlcNAcβ1-3Galβ1-4GlcNAcβ-sp |
| 1259 | *Escherichia coli* O84-deAc |
| 1241 | *Escherichia coli* O81 |
